# RIG-I, MDA5 and TLR3 Synergistically Play an Important Role in Restriction of Dengue Virus Infection

**DOI:** 10.1371/journal.pntd.0000926

**Published:** 2011-01-04

**Authors:** A. M. A. Nasirudeen, Hui Hui Wong, Peiling Thien, Shengli Xu, Kong-Peng Lam, Ding Xiang Liu

**Affiliations:** 1 Institute of Molecular and Cell Biology, Singapore, Singapore; 2 School of Biological Sciences, Nanyang Technological University, Singapore, Singapore; 3 Immunology Group, Bioprocessing Technology Institute, Singapore, Singapore; University of California, Berkeley, United States of America

## Abstract

Dengue virus (DV) infection is one of the most common mosquito-borne viral diseases in the world. The innate immune system is important for the early detection of virus and for mounting a cascade of defense measures which include the production of type 1 interferon (IFN). Hence, a thorough understanding of the innate immune response during DV infection would be essential for our understanding of the DV pathogenesis. A recent application of the microarray to dengue virus type 1 (DV1) infected lung carcinoma cells revealed the increased expression of both extracellular and cytoplasmic pattern recognition receptors; retinoic acid inducible gene-I (RIG-I), melanoma differentiation associated gene-5 (MDA-5) and Toll-like receptor-3 (TLR3). These intracellular RNA sensors were previously reported to sense DV infection in different cells. In this study, we show that they are collectively involved in initiating an effective IFN production against DV. Cells silenced for these genes were highly susceptible to DV infection. RIG-I and MDA5 knockdown HUH-7 cells and TLR3 knockout macrophages were highly susceptible to DV infection. When cells were silenced for only RIG-I and MDA5 (but not TLR3), substantial production of IFN-β was observed upon virus infection and vice versa. High susceptibility to virus infection led to ER-stress induced apoptosis in HUH-7 cells. Collectively, our studies demonstrate that the intracellular RNA virus sensors (RIG-I, MDA5 and TLR3) are activated upon DV infection and are essential for host defense against the virus.

## Author Summary

Dengue fever, dengue haemmorhagic fever and dengue shock syndrome, which are caused by dengue virus infection, are a major public health problem in many parts of the world, especially South East Asia. The investigation of host cell transcriptional changes in response to virus infection using DNA microarray technology has been an area of great interest. In our previous study, we used microarray technology to study expression of individual human genes in relation to dengue virus infection. Most of the genes that were upregulated were type 1 interferon related genes. To gain a better understanding of the innate immune response to dengue virus, we knocked down RIG-I, MDA5 and TLR3 genes in HUH-7 cells. Silencing these genes using siRNA technology resulted in significant increase in viral replication. This increase in viral load induced ER stress leading to apoptosis. This study demonstrates a synergistic role for RIG-I, MDA5 and TLR3 in restricting dengue virus infection.

## Introduction

Pathogen associated molecular patterns (PAMP) trigger innate immunity against pathogens and this response represents the first line of defense against various microorganisms [1]. Double strand RNA (dsRNA), a viral replication intermediate, is sensed by cytoplasmic RNA helicases retinoic acid-inducible gene I (RIG-I) and melanoma differentiation-associated gene 5 (MDA5) as well as by toll-like receptors-3 (TLR3) [2]. TLR3 and RNA helicases interact with different PAMP during the proximal signaling events triggered by the dsRNA. However, these two parallel viral recognition pathways converge at the level of IFN regulatory factor-3 (IRF3). Phosphorylation of IRF3 initiates antiviral responses, including the activation of type I interferon (IFN), interferon stimulating genes (ISGs) and proinflammatory cytokines [3], [4].

While TLR3 is primarily responsible for recognizing viral components such as viral nucleic acid and envelope glycoproteins in the extracellular and endosomal compartments [5], DExD/H box–containing RNA helicases - RIG-I, MDA5 - recognize intracellular dsRNA and they constitute the TLR-independent IFN induction pathway. Although both RIG-I and MDA-5 share high degree of functional and structural homology, they were observed to respond to different dsRNA moieties and RNA viruses. They contain caspase-recruiting domains (CARD) that allow them to interact with Interferon Promoter Stimulated 1 (IPS-1) (otherwise known as Virus-induced Signaling adapter (VISA); mitochondrial antiviral signaling protein (MAVS) or Cardif) [6]. Similar to TLR3, IPS-1 mediates activation of TBK1 and IKKε which in turn activates/phosphorylates IRF3. Phosphorylated IRF3 then homodimerises and translocates to the nucleus [7] to stimulate the expression of type I interferons – IFN-α and IFNβ. IFN-α/β, together with an array of other interferon stimulated genes (ISGs) and cytokines, lead to the establishment of an antiviral state which restricts virus spread in the host cells.

Dengue virus was reported to induce type I IFN even in RIG-I or MDA5 null cells [8]. The same is observed with West Nile virus [9], another Flavivirius. Japanese encephalitis virus [10] and Hepatitis C virus [11], also belonging to the Flavivirdae family, on the other hand, are recognized only by RIG-I. These results suggest that Flaviviruses, despite their common genomic features and replication strategies, are differentially recognized by the host. Despite having the IFN pathway activated in response to viral infection, pathogenic viruses have evolved ways to manipulate the IFN system to favor their survival in the host cell. Reports have shown that DV can antagonize the IFN pathway via their non-structural proteins such as NS2A, NS2B, NS4B and NS5 [12], [13].

A recent microarray analysis of dengue virus type 1 (DV1)-infected lung carcinoma cell line, H1299, showed upregulation of a number of innate immune response genes. In particular, RIG-I, MDA5 and TLR3 were up-regulated more than 8-, 5- and 2-fold respectively [14]. Furthermore, Sumpter and colleagues [11] reported that inactivation of RIG-I in HUH-7 cells resulted in permissiveness of hepatitis C virus (HCV; a flavivirus) RNA replication. Since HUH-7 cells have low basal level of Toll-like receptor 3 (TLR 3) gene expression, this cell line would be a good *in vitro* model system to investigate RIG-I-dependent signaling in DV1 infection [15].

In this study, we present evidence that DV1 infection results in the upregulation of RIG-I, MDA5 and TLR3 expression in HUH-7 cells. This is the first study that shows the role of all three viral RNA sensors – RIG-I, MDA5, TLR3 – in the same cellular system. Previous studies have shown the role of these sensors in different cell lines which may not take into consideration the differences in the genetic make-up of the cell lines. We show, in this study, how RIG-I, MDA5 and TLR3 signaling pathways play a role in DV1-infected cells.

## Materials and Methods

### Cell lines, virus infection and total cellular RNA isolation

Wild type and TLR3 knockout macrophages were kind gifts from Dr. Xu Shengli, Bioprocessing Technology Institute, Singapore. Macrophages, HUH-7 and shRIG-I cells were cultured in DME medium containing 5% fetal bovine serum (FBS) and 1% penicillin and streptomycin antibiotics (PSA). One set of uninfected macrophages, HUH-7 and shRIG-I cells served as a control, while another set was infected with the Singapore strain of dengue type 1 virus at a multiplicity of infection (MOI) of 1 and incubated at 37°C for 2 h. The supernatant was replaced with fresh DME containing 1% FBS, and infected and uninfected cells were harvested after 3 days. Mock represents cells incubated/transfected with cell lysates/vector for a period of time similar to the infected cells. Total RNA was extracted using the Trizol reagent (Invitrogen, USA) and RNA concentrations quantified via UV spectrophotometry at 260 and 280 nm. RNAs with an OD_260 nm_∶OD_280 nm_ absorbance ratio of at least 1.9 with intact ribosomal 28S and 18S RNA bands were used in this study.

### UV inactivation of DV1

DV1 was inactivated by exposing the virus to a UV lamp (wavelength, 254 nm) at a distance of 5 cm for 1 h. UV-inactivation was confirmed by the inoculation of C6/36 cells before use and, in individual experiments, by monitoring the exposed cells for synthesis of viral non-structural protein, NS3, at 72 h. The supernatant fluids from the inoculated cells were also monitored for the presence of infectious virus.

### Mice and TLR3−/− knockout macrophages

TLR3−/− mice have been described previously [16] and maintained in the C57BL/6 background. Wild type C57BL/6 mice were from the Jackson Laboratory (Bar Harbor, Me). Bone marrow-derived macrophages (BMDMs) were generated by culture of bone marrow cells in DMEM containing 20% FCS and 30% L929 conditioned medium (DMEM-C) for 6 days. BMDMs were harvested and tested for purity by flow cytometry with antibodies specific for F4/80 and Mac-1 (BD Pharmingen). The purity of BMDMs was typically 90–95%

### Real-time and semi-quantitative RT-PCR

Total RNA (5 µg) from DV1 infected and uninfected cells were reverse-transcribed. Real-time RT-PCR was carried out for the selected genes using gene-specific primers and the LightCycler-FastStart DNA Master^PLUS^ SYBR Green 1 reaction mix (Roche Molecular Biochemicals, Mannheim, Germany). The LightCycler system was used to monitor the SYBR Green signal at the end of each extension period for 40 cycles. The threshold cycle (C_T_) for each gene of interest and for the GAPDH housekeeping gene, and the difference between their C_T_ values (ΔC_T_) were determined. The relative expression values (2^−ΔΔCT^) between uninfected and infected samples for the selected genes were determined by using the uninfected sample as the reference with its ΔC_T_ value subtracted from the ΔC_T_ value of the infected sample (i.e. ΔΔC_T_). Relative fold difference values shown in figures are average of at least two independent experimental results.

A two-step semi-quantitative RT-PCR method was used to measure gene expression in the DV1 infected and uninfected samples. Random hexamers (Qiagen Inc.) was used as primer in the first step of cDNA synthesis. Total RNA (5 µg) was combined with 2 µl of random hexamers, 200 µM dNTPs and H_2_0 and preheated at 65°C for 10 min to denature secondary structures. The mixture was then cooled rapidly in ice and then 5 µl 5×RT Buffer, 10 mM DTT, 0.5 µl RNAse inhibitor (Roche), 1.0 µl (10 mM) dNTP and 200 U reverse transcriptase (Roche) were added for a total volume of 20 µl. After pulse spinning, the RT mix was incubated at 43°C for 90 min and then stopped by heating at 95°C for 5 min. The cDNA stock was stored at −20°C. The yield of cDNA was measured according to the PCR signal generated from the internal standard house-keeping gene GAPDH amplified 30 cycles with 1 µl of the cDNA solution. Gene-specific PCR amplifications were carried out by adding 5 µl of 10 x PCR buffer, 1 µl (5 U/µl) Taq Polymerase, 1.0 µl (10 mM) dNTP, 1 µl of each primer, 1.5 µl (50 mM) MgCl_2_, 3 µl of the first strand cDNA and double-distilled water to 50 µl. The PCR products were loaded onto ethidium bromide-stained 1% agarose gels. A 100 bp DNA ladder molecular weight marker (Fermentas) was run on every gel to confirm expected molecular weight of the amplification product.

The Primer pairs used were: OAS2: sense, TGAGAGCAATGGGAAATGGG, anti-sense, AGGTATTCCTGGATAAACCAACCC; RIG-1: sense, TGTGGGCAATGTCATCAAAA, anti-sense, GAAGCACTTGCTACCTCTTGC; MDA5: sense, GGCACCATGGGAAGTGATT, anti-sense, ATTTGGTAAGGCCTGAGCTG; IFNβ: sense, CTCTCCTGTTGTGCTTCTCC, anti-sense, GTCAAAGTTCATCCTGTCCTTG; ISG15: sense, TGGTGGACAAATGCGACGAA, anti-sense, CAGGCGCAGATTCATGAAC; ISG56: sense, TCTCAGAGGAGCCTGGCTAAG, anti-sense, CCACACTGTATTTGGTGTCTAGG; XBP1: sense, CTGGAAAGCAAGTGGTAGA, anti-sense, CTGGGTCCTTCTGGGTAGAC; GAPDH: sense, GACAACTTTGGTATCGTGGAA, anti-sense, CCAGGAAATGAGCTTGACA.

### Flow cytometry and statistical analysis

TUNEL assay. TdT-mediated dUTP-biotin nick-end labeling (TUNEL) was performed using ApoAlert DNA Fragmentation Assay kit (Clontech) according to the manufacturer's instructions. Briefly, the cells were fixed with 4% formaldehyde/PBS and resuspended in 0.2% Triton X–100 and incubated on ice for 5 min. The cells were labeled by adding 50 µl TUNEL mix. The samples were then resuspended in PBS prior to flow cytometry (FACS Calibur; Becton-Dickinson, San Jose, CA) and results displayed using WinMDI 2.8 software program.

Subgenomic content. To measure subgenomic content, cells were fixed with 70% ice-cold ethanol and stained with 50 µg/ml propidium iodide (PI) containing RNase and subgenomic content was evaluated by a flow cytometer.

Statistical analysis. Error bars in figures represent data expressed as the mean ± S.D. of at least three independent experiments. Z-test for two simple means was used to calculate P-value.

### Immunoblotting and antibodies

Cells were harvested up to 72 hpi, and lysed in RIPA buffer containing protease inhibitors. The lysates were subjected to immunoblotting using primary and HRP-conjugated secondary antibodies followed by the ECL-Plus chemiluminescence substrates (Amersham). Mouse monoclonal DV1 E and NS3 antibodies used were prepared in-house. Anti RIG-I and anti-MDA5 (Axxora); anti-actin (Santa Cruz); anti-calreticulin (BD Transduction Laboratories); anti-IRF3 (Santa Cruz) and anti-myc (Sigma Aldrich).

### Generation of RIG-I knock-down cells, plasmid constructs and transfection of polyI:C

As shRNA target in the RIG-I sequence, 5′-AATTCATCAGAGATAGTCA -3′ was chosen HUH-7 cells were transfected with shRNA constructs using Lipofectamine 2000 reagents (Invitrogen). Clones were selected in the presence of G418 and screened for reduced RIG-I expression. For over-expression studies, RIG-I and MDA5 coding regions were cloned into a mammalian expression vector. The identities of the clones were confirmed by DNA sequencing. Synthetic dsRNA polyI:C was purchased from Sigma Aldrich. Cells were transfected with 1 µg/ml polyI:C for various times using Lipofectamine 2000 (Invitrogen) according to the manufacturer's instructions.

### Small interfering RNA (siRNA) assay

For gene silencing of RIG-I: sense, CAGAAGAUCUUGAGGAUAAUU was used. siTLR3 was purchased from Sigma Aldrich. HUH-7 cells (1×10^5^ per well) were plated in six-well plates. At 24 h after incubation, cells were washed, replenished with medium without serum and transfected, at 40–60% of confluency, with specific siRNA or control siRNA by using a siRNA transfection reagent (Lipofectamine RNAi Max reagents, Invitrogen) according to the manufacturer's instructions. After 8 h incubation at 37°C, the liposome suspension was removed and complete culture medium was added. After 24–48 h, cells were infected with DV1 and then harvested at 48 h for analysis. Cells that were untreated or treated with control siRNA served as controls.

### IRF3-dimerisation assay

Cells were lysed in buffer containing 50 mM Tris HCl (pH 7.4), 150 mM NaCl, 1 mM EDTA, 1% NP-40, protease inhibitors and phosphatase inhibitors for 30 min at 4°C. Proteins were separated by electrophoresis in 8% non-denaturing polyacrylamide gels containing 1% sodium deoxycolate in the cathode buffer. IRF3 monomers and dimers were detected by Western blot using polyclonal antibodies against the full-length IRF3 (Santa Cruz).

### Immunofluorescence

Cells were grown on 24-well plates. After infection with DV1 for the indicated times, cells were fixed in cold methanol for 10 min, washed in PBS and blocked in normal goat serum for 1 h. DV1 E antibody was then applied for 1 h followed by FITC-conjugated secondary for another 1 h. The cells were then washed 4 times with PBS and examined under a fluorescence microscope or quantified using a flow cytometer.

### Enzyme-linked immunosorbent assay (ELISA)

Cells transiently transfected or cells infected with virus were cultured for up to 48 h. IFN-β in the supernatants was measured using VeriKine Human IFN-β ELISA kit (PBL Biomedical Laboratories) according to the manufacturer's instructions.

## Results

### RIG-I and MDA5 knockdown in HUH-7 cells results in enhancement of DV1 replication

**Figure 1:**
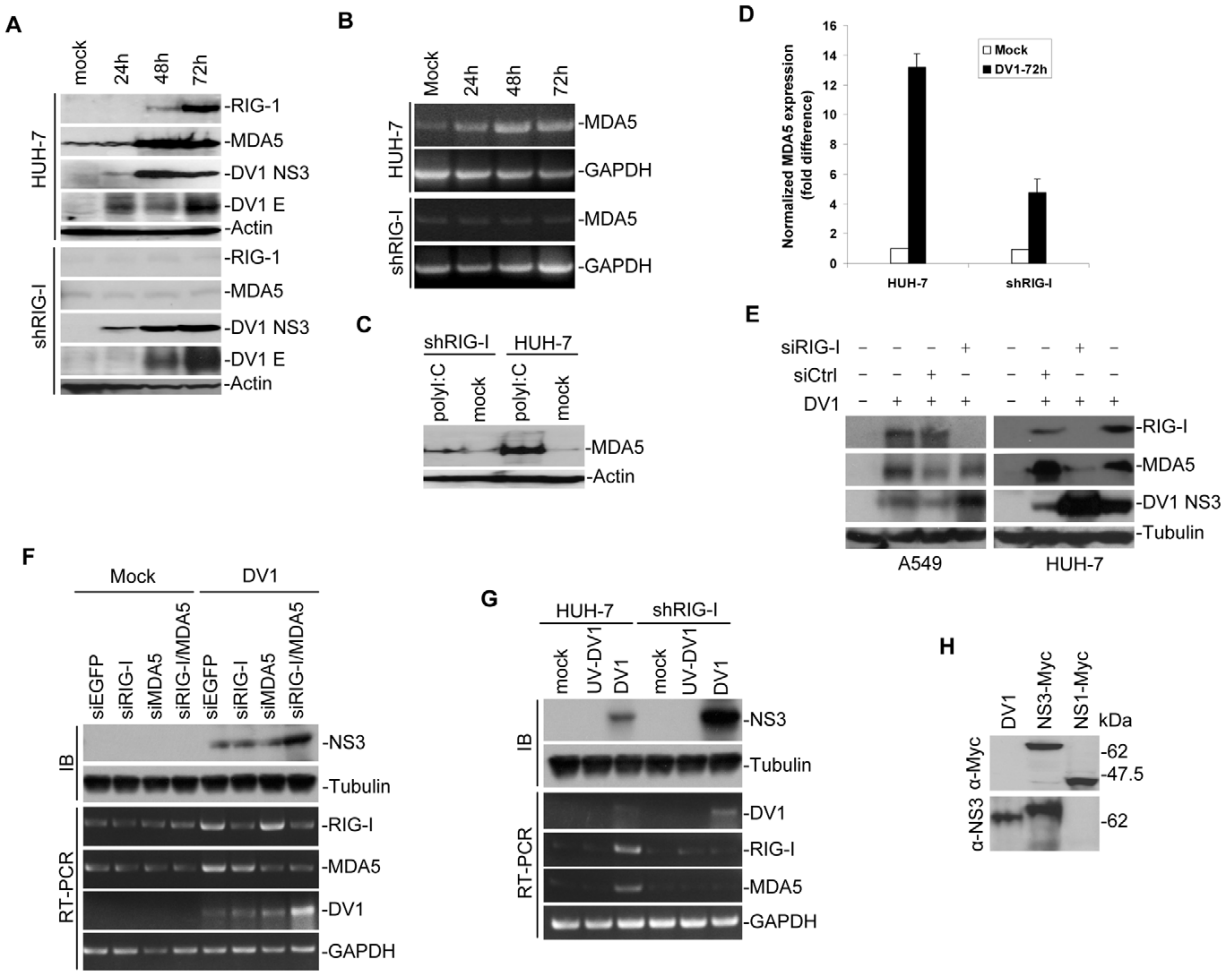
DV1 infection of HUH-7 and shRIG-I cells. (A) Whole cell lysate from DV1-infected cells were subjected to Western blot analysis and probed for the antibodies indicated and visualized by enhanced chemiluminescence. The nitrocellulose membranes were then reprobed for β-actin (loading control). (B) Qualitative RT-PCR for MDA5 mRNA level in DV1-infected cells from different time points. Total RNA was isolated and subjected to RT-PCR analysis. GAPDH was used as a control for equal RNA templates. (C) Uninfected shRIG-I and HUH-7 cells were stimulated with synthetic polyI:C. Cell lysates were assessed after 24 h of stimulation by Western blot for MDA5. β-actin was used as a control for equal loading of cell lysates. (D) Real-time RT-PCR analysis of MDA5 expression in mock and DV1-infected cells. Total RNA was isolated, used for cDNA preparation and subjected to real-time RT-PCR analysis. Bar histograms show the average difference in gene expression between mock and DV1-infected cells based on at least two independent experiments. (E) siRNA silencing technique was used to silence RIG-I gene. In HUH-7 cells, and not in A549 cells, RIG-I silencing co-induced silencing of MDA5 gene. (F) A549 cells were transfected with siRNA for EGFP, RIG-I and/or MDA5. Whole cell lysate from mock and DV1-infected A549 cells were subjected to immunoblotting (IB) and RT-PCR analysis. Results show that DV1 infection is significantly higher in cell line deficient for both RIG-I and MDA5. (G) Whole cell lysate from HUH-7 and shRIG-I cells infected with either wild type or UV-treated DV1 virus were subjected to immunoblotting (IB) and RT-PCR analysis. DV1 was UV-inactivated by exposing the virus to a UV-lamp (wavelength, 254 nm) at a distance of 5 cm for 1 hour. Results show that UV-treated DV1 infection did not activate RIG-I and MDA5. (H) Specificity of monoclonal NS3 antibody. Myc-tagged DV1 NS1 and NS3 constructs were transfected into HUH-7 cells. Cell lysates were harvested 24 h later and used to test specificity of monoclonal NS3 antibody. DV1-infected HUH-7 cell lysate was used as a control. Anti-NS3 antibody was found to be specific for DV1 NS3 protein.

To study if RIG-I influences DV1 infection and understand the relationship between innate antiviral responses and virus replication, we used short-hairpin RNA interference (shRNA) technology to knockdown RIG-I in HUH-7 cells. A clonal population of stably transfected cells that showed decreased expression for RIG-I was selected. RIG-I knock-down cells (shRIG-I) were infected with DV1 for up to 72 hours and subjected to immunoblotting analysis. RIG-I expression increased over time in DV1-infected wild type HUH-7 cells and there was minimal RIG-I up-regulation in the shRIG-I cells (Fig. 1A). Since Loo and colleagues [8] demonstrated that dengue virus type 2 triggered both RIG-I and MDA5, we probed for MDA5 expression in DV1-infected cells. Minimal MDA5 expression was observed in DV1-infected shRIG-I cells while increased MDA5 expression was observed in HUH-7 cells (Fig. 1A). Although MDA5 seems to be upregulated faster than RIG-I in HUH-7 cells, the basal level (see mock infection) of the two proteins are not the same. It appears that HUH-7 cells have a higher basal level of MDA5 compared with RIG-I.

Mock-infected HUH-7 cells showed expression of MDA5, whereas shRIG-I cells did not. To investigate if this MDA5 down regulation also occurred at the transcriptional level, RT-PCR was performed on RNA extracted from DV1-infected cells. MDA5 expression at the mRNA level was visibly low in shRIG-I cells as compared to HUH-7 cells (Fig. 1B). To define if this MDA5 down regulation was due to clonal specificity, another clone of shRIG-I cells were infected with DV1 and probed for MDA5 expression. Minimal MDA5 expression was observed in the second clone as well (data not shown). It was important to ensure that this phenomenon is not due to DV1 infection, which may not be activating or could be inhibiting MDA5 expression in these cells. A synthetic analog of viral dsRNA, polyinosine-polycytidylic acid (polyI:C), that can bind and activate MDA5 expression was used to investigate the level of MDA5 expression in shRIG-I and HUH-7 cells. MDA5 expression in shRIG-I cells was reduced even with polyI:C transfection (Fig. 1C). Quantitative RT-PCR showed that the MDA5 expression was decreased 8-fold in shRIG-I cells as compared to HUH-7 cells upon DV1 infection (Fig. 1D). To understand if MDA5 expression was related to RIG-I expression in mammalian cells, we transfected RIG-I siRNA (which had a different target sequence to that of RIG-I shRNA) in A549 and HUH-7 cells. We observed that transfection of RIG-I siRNA significantly knocked down both RIG-I and MDA5 in HUH-7 cells but only RIG-I in A549 cells (Fig. 1E). Knock down of RIG-I in both A549 and HUH-7 cells showed an increase in DV1 propagation. Since the sequences of RIG-I shRNA and RIG-I siRNA are different, it is thus unlikely that these siRNAs knocked down a common off-target gene such as MDA5. To show that this response is not cell-type specific, A549 cells were transfected with siRIG-I or/and siMDA5. We observed that knockdown of both RIG-I and MDA5 showed an increase in DV1 propagation as noticed in shRIG-I cells (Fig. 1F). This result confirms that the observations in HUH-7 and shRIG-I cells are not cell-type specific. To rule out the possibility that contamination of cellular nucleic acids in the virus preparations that could activate RIG-I and MDA5, the same virus preparations were UV-treated and used to infect HUH-7 and shRIG-I cells. Infection of cells with UV-treated DV1 did not result in any significant increase in RIG-I or MDA5 expression (Fig.1G).

**Figure 2:**
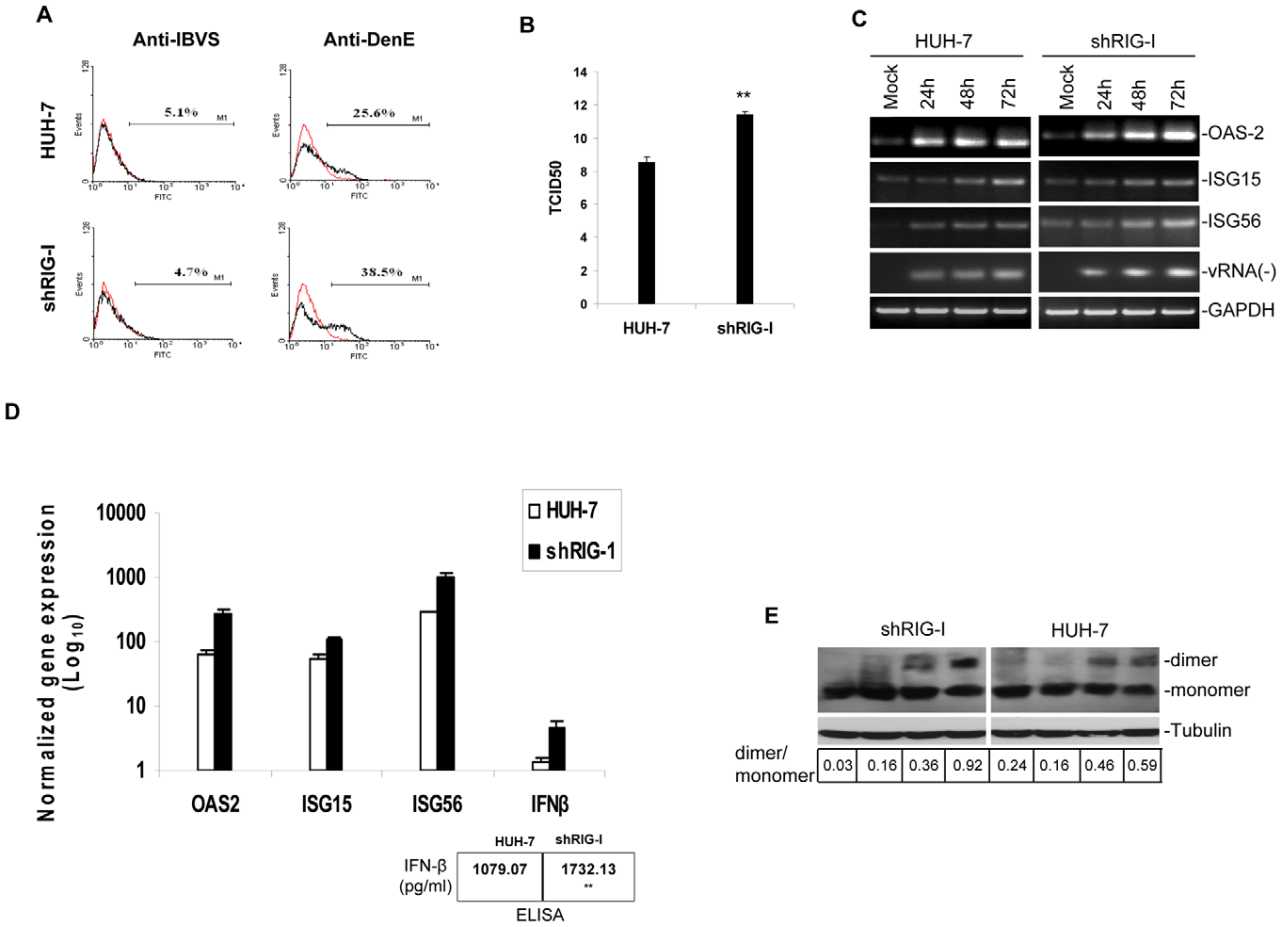
DV1 replication and propagation in HUH-7 and shRIG-I cells. (A) Flow cytometric analysis showed enhanced DV1 infection in shRIG-I cells as compared to HUH-7 cells. Cells were stained with anti-Dengue E or IBV S (an isotype control) antibodies and analyzed by flow cytometry. (B) Tissue Culture Infectious Dose 50 (TCID50) assay. Virus titers in 50% tissue culture infectious doses (TCID_50_)/ml were determined according to Reed and Muench [45]. Symbols indicate significantly different from infected HUH-7 (**P<0.01). (C) Qualitative RT-PCR detection of IFN-related gene expression. Cells grown in 6-well plates were infected with DV1 for up to 72 h. Total RNA was extracted, used for cDNA synthesis and subjected to RT-PCR analysis. GAPDH was used as a control for equal RNA templates. (D) Real-time RT-PCR analysis of gene expression in mock and DV1-infected cells. Total RNA was isolated, used for cDNA preparation and subjected to real-time RT-PCR analysis. Bar histograms show the average difference in gene expression between mock and DV1-infected cells based on at least two independent experiments. IFN-β production was assayed using ELISA (lower panel). Symbols indicate significantly different from infected HUH-7 cells (**P<0.01). (E) Total cellular protein was extracted and analyzed by immunoblot. IRF3 dimerization was assessed by native PAGE with anti-IRF3 antibody as a probe. Tubulin protein level was assessed by SDS PAGE to ensure equal loading of cell lysate. Arrows indicate IRF3 dimer and monomer. Row below figure shows ratio of dimer: monomer analysed by densitometry.

DV1 infection of HUH-7 and shRIG-I cells over a period of 72 hours showed that the latter was highly permissive for DV1 propagation as noted by high levels of viral proteins (Fig. 1A). Antibodies against DV1 E (structural) and NS3 (non-structural) proteins were used to show effective and efficient DV1 propagation in shRIG-I cells. Furthermore, knockdown of RIG-I, using siRNA duplexes, in A549 and HUH-7 cells also showed an increase in DV1 propagation (Fig. 1E). HUH-7 and shRIG-I cells were infected with DV1 and fixed at 48 and 72 h for immunofluorescence assay using a monoclonal antibody directed against DV1 E protein. Enhanced fluorescence intensity was observed in DV1-infected shRIG-I cells as compared to infected HUH-7 cells (data not shown). This fluorescence intensity was quantified by flow cytometric analysis using monoclonal antibody directed against DV1 E protein. A significant increase in DV1-infected cells in shRIG-I cells as compared to infection in HUH-7 cells was observed (Fig. 2A). To define if this enhancement effect was due to increase in viral replication, quantitative RT-PCR (real-time RT-PCR) for negative strand DV1 RNA level, which is indicative of viral replication, was performed. Real-time RT-PCR showed more than 3-fold increase in negative strand DV1 RNA level in shRIG-I cells compared to HUH-7 cells (data not shown). An immunofluorescent-based TCID50 assay was used to titrate the amount of infectious particle in HUH-7 and shRIG-I cells (Fig. 2B). DV1 propagated significantly higher in shRIG-I cells than in HUH-7 cells.

### DV1 activates innate immune-responsive genes in RIG-I- and MDA5-knockdown cells

Recently, Loo and colleagues [8] demonstrated that RIG-I and MDA5 are equally essential for IFN-β production induced in response to infection with dengue virus. To determine whether DV1 induces IFN-β promoter activity in HUH-7 and shRIG-I cells through a RIG-I and MDA5-dependent pathway, we infected these cells with DV1 for up to 72 h. Total RNA extracted from DV1-infected cells were subjected to qualitative (Fig. 2C) and quantitative (Fig. 2D) RT-PCR to detect mRNA levels of 2′,5′-oligoadenylate synthetase 2 (OAS2), interferon stimulated gene (ISG) 15, ISG56 and IFN-β genes. These genes were chosen for detection because a recent microarray analysis of DV1-infected H1299 cells (human non-small lung cancer cells) showed increased expression of OAS2, ISG15 and ISG56 [14]. RT-PCR assays showed a significant increase in mRNA levels of IFN-β and IFN stimulated genes (OAS2, ISG15 and ISG56) in DV1-infected shRIG-I cells as compared to infected HUH-7 cells. Qualitative RT-PCR for negative strand viral RNA was performed to show level of virus replication in the infected cell lines (Fig. 2C). Virus replication was much pronounced in shRIG-I cells. Quantitative RT-PCR showed at least 200-, 50-, 700- and 4-fold difference for OAS2, ISG15, ISG56 and IFN-β gene expression respectively between DV1- infected HUH-7 and shRIG-I cells (Fig. 2D). IFN-β production was assayed using ELISA. DV1-infected shRIG-I cells produced more significant levels of IFN-β compared with DV1-infected HUH-7 cells (Fig. 2D; lower panel).

When dsRNA is sensed in the cytoplasm, RIG-I and/or MDA5 recruit the adaptor protein IPS-1 [17], [18] resulting in the downstream activation (dimerization) and nuclear localization of IRF3 which acts as a transcriptional factor for IFN-α/β expression. IRF3 dimerization was assessed by native PAGE with anti-IRF3 antibody as a probe. Figure 2D shows stronger IRF3 dimer formation in DV1-infected shRIG-I cells compared to infected HUH-7 cells. Ratio of IRF3 dimer/monomer shows greater dimer formation in shRIG-I cells (0.92) as compared to HUH-7 cells (0.59) at 72 h. Increased permissiveness for DV1 infection in shRIG-I cells could have lead to induction of other stimulators of IRF3, such as TLR3. These results, collectively, show that factors other than RIG-I/MDA5 may play a role in DV1-induced immunity.

### Cellular response to higher dengue virus replication in shRIG-I cells

**Figure 3:**
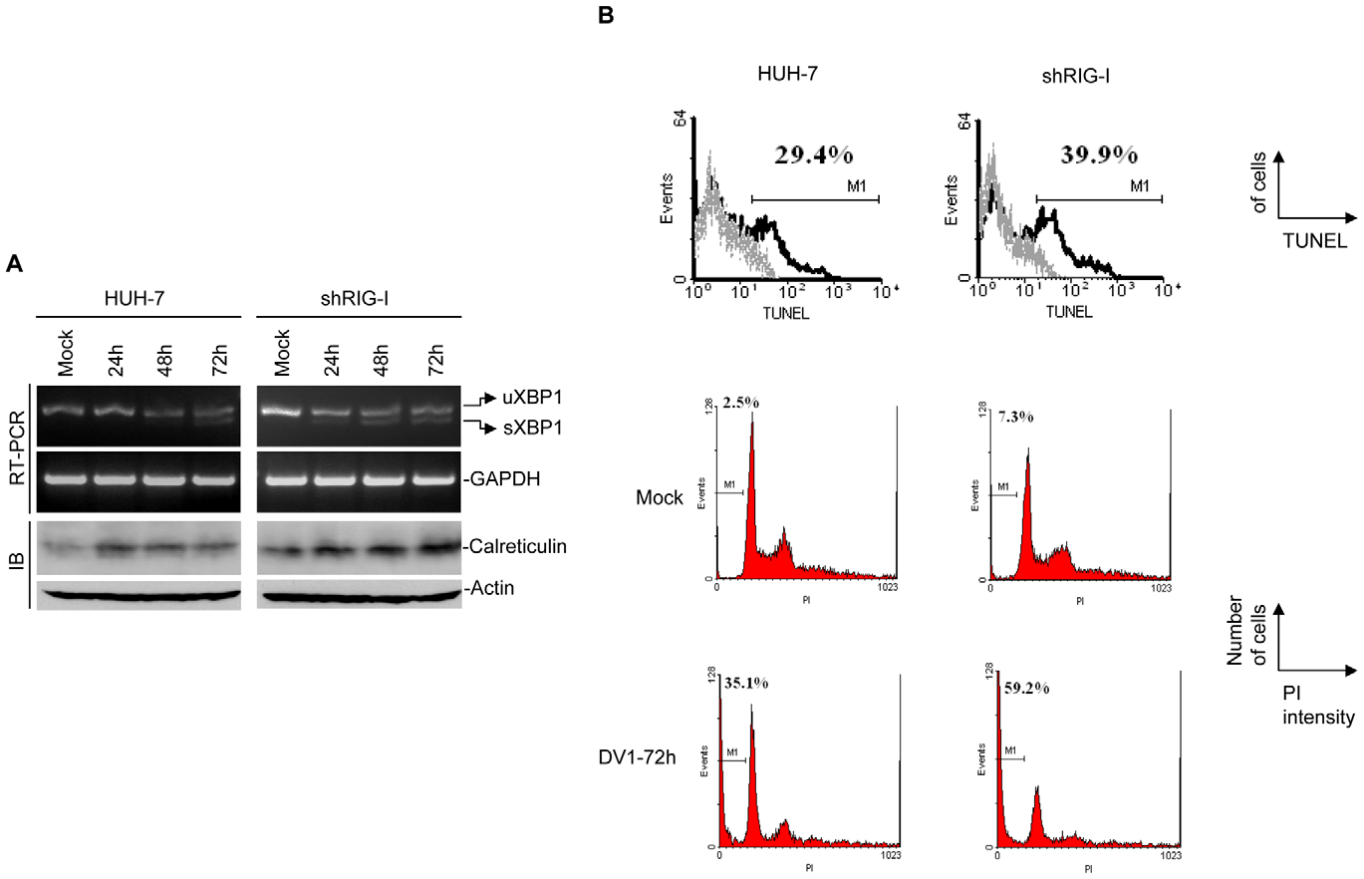
Endoplasmic reticulum (ER) stress-induced expression of XBP1 mRNA and calreticulin protein expression. (A, upper panel) XBP1 splicing in DV1-infected cells. Total RNA samples were prepared and RT-PCR analysis was performed. sXBP1, spliced form of XBP1; uXBP1, unspliced form of XBP1. (A, lower panel) Analysis of calreticulin expression in DV1-infected cells. Whole cell lysate from DV1-infected cells were subjected to Western blot analysis and probed for the calreticulin and visualized by enhanced chemiluminescence. The nitrocellulose membranes were then reprobed for β-actin (loading control). Note increase in calreticulin expression in DV1-infected shRIG-I cells. (B, upper panel) *In situ* DNA fragmentation analysis (TUNEL) of mock and DV1-infected cells was carried out by flow cytometry. (B, lower panel) Flow cytometric analysis of DNA content by PI staining: DV1-infected HUH-7 and shRIG-I cells were analyzed after 72 hpi. The PI uptake rate was determined by flow cytometry and analyzed using WinMDI 2.7 software. Percentage of cells in sub-G1 (M1 region), indicative of cell death, in each sample is shown. DV1-infected shRIG-I cells showed significant increase in sub-G1 content.

Since shRIG-I cells are highly permissive for DV1 replication, this might lead to accumulation of viral RNA and perhaps an overload of the cellular protein synthesis machinery triggering host response to the infection [19]. Viral protein overload in the endoplasmic reticulum could result in unfolded protein response (also known as ER stress) and activation of apoptotic cell death in DV1-infected cells. We investigated if this proliferation of viral antigens in shRIG-I cell would lead to increased ER stress and eventually apoptosis/cell death. ER stress leading to apoptosis in dengue virus-infected cells has been reported previously [20]. As a signal of ER stress, X box binding protein 1 (XBP1) gene expression was detected. XBP-1 is up-regulated as part of the endoplasmic reticulum (ER) stress response, the unfolded protein response (UPR) [21]. During ER stress, IRE-I/XBP1 pathway is activated by cleavage of a 26-nucleotide intron from unspliced XBP1 (uXBP1) mRNA resulting in XBP1 in its mature form (sXBP1) [20]. Infection of shRIG-I and HUH-7 cells by DV1 resulted in splicing of XBP1 (Fig. 3A). Splicing of XBP1 in DV1-infected shRIG-I cells occurs as early as 24 hpi as compared to 48 hpi in infected HUH-7 cells. Our results conform to that of Umareddy *et al.,* [22] whereby the authors showed that XBP1 is spliced upon dengue virus replication.

Since most DV1 proteins are localized on the luminal side of the ER membrane prior to cleavage and processing into mature forms, we investigated ER stress by also detecting the expression of calreticulin, a multifunctional, multi-compartmental protein most abundant in the ER lumen. Calreticulin interacts and assists in the folding of various glycoproteins, including viral proteins [23]. The DV1 envelope protein which is produced in large quantities during infection is an N-linked glycoprotein [24]. More pronounced increase in calreticulin protein expression was observed in DV1-infected shRIG-I cells as compared to HUH-7 cells over a period of 72 hpi (Fig. 3A). Taken together, these results show that ER stress is more profound in shRIG-I cells due to increased permissiveness to DV1 replication.

Apoptosis/cell death is the final outcome of dengue virus infections [25]. Although UPR is necessary for cell survival and viral replication, prolonged UPR can lead to cell death [22]. Furthermore, activation of RIG-I upon virus infection had been reported to activate the apoptotic cascade [26]. Interferon-β promoter stimulator-1 and IRF-3 were shown to be required for efficient apoptosis following reovirus infection, suggesting a common mechanism of antiviral cytokine induction and activation of the cell death response [26]. Since DV1 infection of HUH-7 and shRIG-I cells showed activation of UPR, IRF-3 dimer formation and IFN- β activation, cell death induced by DV1 infection in HUH-7 and shRIG-I cells was investigated. DNA fragmentation, a hallmark of apoptosis, was observed in both DV1-infected HUH-7 and shRIG-I cells in varying degrees (Fig. 3B). *In situ* DNA fragmentation was investigated using TUNEL assay, which relies on the specific binding of terminal deoxynucleotidyl transferase (TdT) to exposed 3′-OH ends of the fragmented DNA. The signal is then amplified by avidin-peroxidase, enabling detection of *in situ* DNA fragmentation by flow cytometry. The results of this assay on DV1-infected cells showed an approximately 10% increase in cell death in shRIG-I cells as compared with HUH-7 cells after 72 hpi (Fig. 3B, upper panel). Cell death in DV1-infected cells was further investigated by analysis of sub-G1/DNA content, indicative of cell death, in HUH-7 and shRIG-I cells. Analysis of the changes in the DNA content distribution showed 20% more sub-G1 DNA in DV1-infected shRIG-I cells as compared to DV1-infected HUH-7 cells (Fig. 3B, lower panel).

### RIG-I and MDA5 synergistically mediate type 1 IFN response

**Figure 4:**
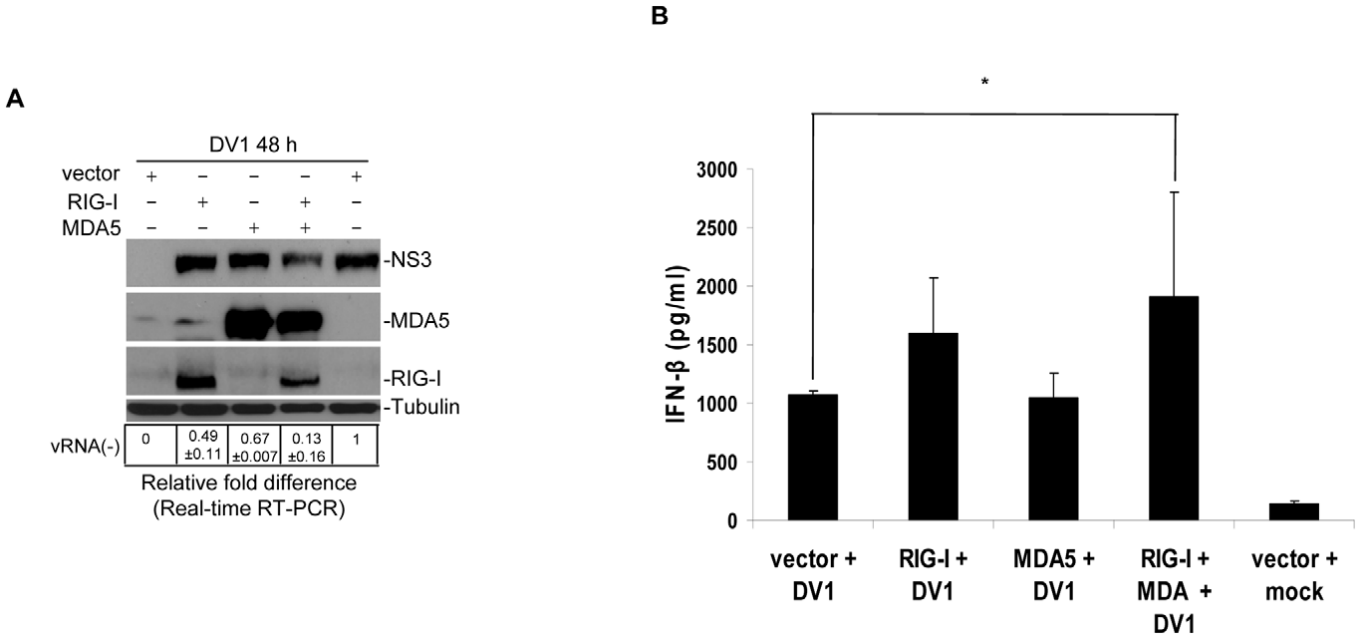
Over-expression of RIG-I and MDA5 in wild type HUH-7 cells. (A) RIG-I and MDA5 constructs were over-expressed in HUH-7 cells and lysates harvested 48 hours post infection. Immunoblotting for antibodies stated on the left shows a decrease in viral antigen in cells double transfected with both plasmids. Real-time RT-PCR analysis of gene expression in mock and RIG-I and/or MDA5 transfected cells. Total RNA was isolated, used for cDNA preparation and subjected to real-time RT-PCR analysis. Rows below figure show negative strand viral RNA. (B) IFN-β production was assayed using ELISA. Symbols indicate significantly different from vector transfected and infected cells (*P<0.05).

To investigate if RIG-I, MDA5 or both helicases influence the initiation of IFN response and to understand their antiviral potencies, we overexpressed these genes in HUH-7 cells. Upon infection with DV1 for up to 48 h, less DV1 propagation was observed in cells transfected with both helicases (Fig. 4A). Real-time RT-PCR showed more than 5-fold decrease in negative strand DV1 RNA level in cells transfected with both RIG-I and MDA5 (0.13) compared to infected HUH-7 cells (1.0). Significant increase in IFN-β gene expression was also noted in infected cells overexpressed with both RIG-I and MDA5 compared to infected HUH-7 cells (Fig. 4B). When transfected individually, RIG-I and MDA5 were not able to efficiently suppress DV1 replication but induced elevated amounts of IFN-β expression. These results clearly show that RIG-I and MDA5 synergistically mediate an antiviral response during DV1 infection.

### Activation of TLR3 in DV1-infected cell lines

**Figure 5:**
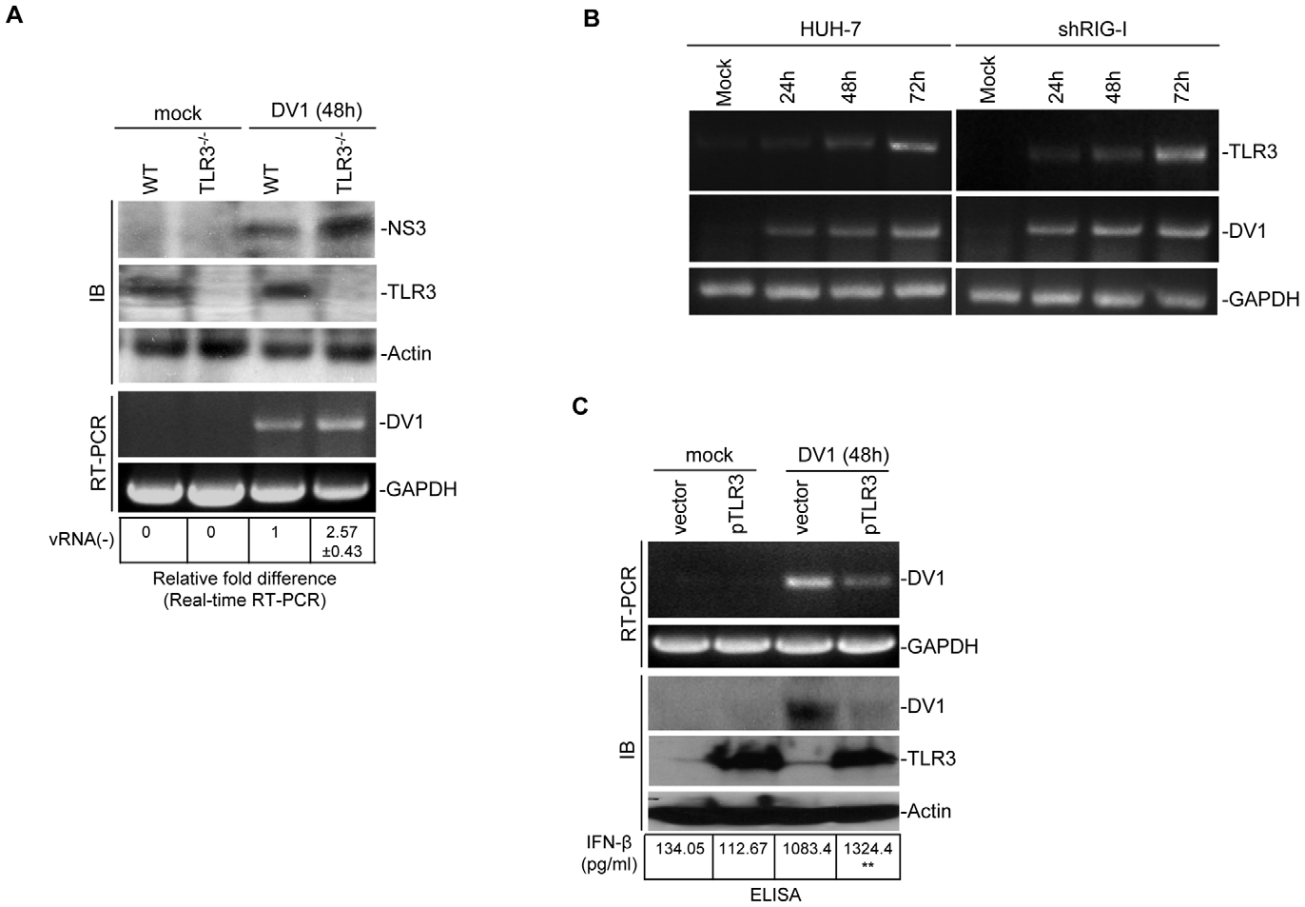
TLR3 knock-out macrophages. Wild type and TLR3 knock-out macrophages were infected with DV1. (A) Cell lysates and total RNA were collected for analysis. Immunoblotting (IB) and RT-PCR analysis show that DV1 infection is significantly higher in cell line deficient for TLR3. Row below figure shows negative strand DV1 RNA level after 48 hours post-infection of HUH-7 and shRIG-I cells as evaluated by quantitative real-time RT-PCR. (B) HUH-7 and shRIG-I cells were infected with DV1 and total RNA was isolated, used for cDNA preparation and subjected to RT-PCR analysis. Note increase in TLR3 expression in shRIG-I cells. (C) TLR3 construct was transfected into HUH-7 cells to over-express the protein. Immunoblotting (IB) and RT-PCR analysis show decreased DV1 infection when TLR3 is over-expressed. IFN-β production was also increased in cells over-expressed with TLR3 and infected with DV1 (row below figure). Symbols indicate significantly different from vector transfected and infected cells (**P<0.01).

Inspite of knocking down RIG-I and MDA5, the level of IFN-β produced seems to increase upon DV1 infection in shRIG-I cells. Furthermore, an increase in cytokine production was noted (Fig. 2C) and an increase in IRF3 dimerization was also observed in native PAGE analysis (Fig. 2E). This phenomenon could be due to an increase in virus replication in the knock down cell line or due to other intracellular receptors such as Toll-like receptor 3 (TLR3) that recognize double-stranded RNA synthesized during DV1 replication. TLR3 has been shown to recognize double-stranded RNA [27] and RIG-I/MDA5 and TLR3 signaling pathways converge to phosphorylate IRF3 [28]. We investigated if TLR3 could recognize DV1 infection. Since the basal level of TLR3 is low in HUH-7 cells, we used wild type (WT) and TLR3-knockout (TLR3ko) macrophages. Furthermore, TLR3 is abundant on macrophages [29]. While this work was in progress, Tsai *et al.,* [30] reported that TLR3 in HEK293 cells recognizes dengue virus type 2 and induces cytokine production. In our study, WT and TLR3ko macrophages were infected with DV1 for 48 h and cell lysate and RNA were collected for analysis. Immunoblotting analysis and both semi-quantitative and real-time RT-PCR showed that DV1 virus propagation was more efficient in TLR3ko macrophages (Fig. 5A). Negative strand viral RNA, indicative of DV1 replication, was quantitated using real-time RT-PCR. Approximately 2.5 fold increase in viral RNA was noted in DV1-infected shRIG-I cells as compared to infected HUH-7 cells. These experiments show that TLR3 recognizes DV1 double stranded RNA intermediate.

DV1 infected HUH-7 and shRIG-I cells showed increase in TLR3 expression over 72 h (Fig. 5B). This result was confirmed by real time RT-PCR analysis (data not shown). To determine if TLR3 could modulate DV1 replication, we overexpressed TLR3 in HUH-7 cells and infected with DV1 for up to 48 h. Semi-quantitative RT-PCR and immunoblotting show that over-expression of TLR3 plasmid greatly inhibited DV1 replication in cells (Fig. 5C). IFN-β gene expression was quantified for the same set of experiments. Cells over-expressing TLR3 showed significant increase in IFN-β production (Fig. 5C).

**Figure 6:**
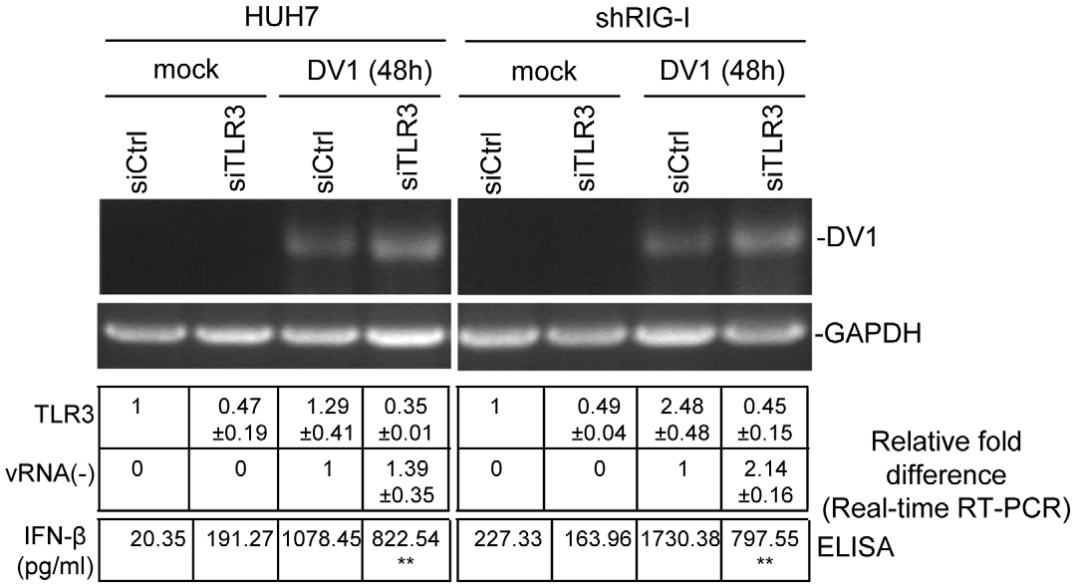
siRNA silencing of TLR3 in HUH7 and shRIG-I cell lines. siTLR3 was transfected in HUH7 and shRIG-I cells and total RNA was tested for DV1 negative strand RNA by semi-quantitative RT-PCR. Rows below figure shows quantitative analysis of DV1 negative strand RNA, TLR3 and IFN-β production in infected HUH7 and shRIG-I cells. Symbols indicate significantly different from vector transfected and infected cells (**P<0.01).

To gain deeper understanding of the role of TLR3 in DV1 infection, siRNA silencing of TLR3 in HUH-7 and shRIG-I cells was carried out. HUH-7 and shRIG-I cells were transfected with siTLR3 and infected with DV1 for 48 h. Since the basal level of HUH-7 is low, real-time RT-PCR analysis was used to determine TLR3 knock down efficiency in cells (Fig. 6). Control siRNA transfected cells showed increase in TLR3 expression upon DV1 infection as compared to siTLR3 transfected cells. Semi-quantitative and real-time RT-PCR analysis for DV1 negative strand RNA showed increase in DV1 replication in siTLR3 transfected cells with reference to GAPDH expression (Fig. 6). IFN-β production was quantified by ELISA for the same set of experiments, showing significant increase in IFN-β expression in shRIG-I cells transfected with siTLR3 and infected with DV1, as compared to control siRNA transfected and infected cells for the same (Fig. 6). Quantitative real-time RT-PCR for negative strand viral RNA shows a higher fold increase in DV1 replication in shRIG-I cells (2.14) as compared to HUH-7 cells (1.39). These data are evidence that DV1 RNA is recognized by TLR3 and such recognition modulates DV1 replication by eliciting IFN-β production.

## Discussion

Innate immune defenses are the first line of host anti-pathogen mechanisms. Among them, type 1 IFNs play a critical role in host defense against virus infection. IFN pathway is triggered through host recognition of viral macromolecular motifs also known as pathogen-associated molecular patterns (PAMP). In this study, we show that IFN-β production in response to DV1 was enhanced in RIG-I and MDA5 down-regulated cells. Knockdown of RIG-I and MDA5 enhanced cellular permissiveness to DV1 RNA replication and also increased virus propagation. Consequently, higher expression of IFN-related and stimulated genes, such as OAS2, ISG15, ISG56, as well as strong activation of IRF3 was observed in the cell line. Analysis of ER stress responses, such as splicing of XBP1 and activation of calreticulin, also showed earlier splicing of XBP1 and accumulation of more calreticulin in RIG-I- and MDA5-knockdown cells. Prolonged ER stress, probably due to increase in viral load, resulted in stronger induction of apoptosis in these cells. Further analysis of the functional roles of RIG-I and MDA5 demonstrated that RIG-I and MDA5 synergistically suppressed DV1 infection with induction of IFN-β production in wild type HUH-7 cells over-expressing the two helicases. Meanwhile, using siRNA against TLR3 showed that down-regulation of TLR3 resulted in higher DV1 infection, and over-expression of TLR3 inhibited DV1 infection significantly by inducing high levels of IFN-β production. When siTLR3 was transfected in shRIG-I cells (down regulated for RIG-I and MDA5), increased permissiveness to DV1 infection was observed. TLR3 knockout macrophages also showed increased permissiveness to DV1 infection than wild type cells. Collectively, our results showed that RIG-I, MDA5 and TLR3 are able to recognize DV1 infection and establish a strong antiviral state in these cells.

Available evidence reveals that RIG-I and MDA5 play differential roles in host antiviral defence against viral infections. RIG-I was shown to respond to RNA bearing a triphosphate at their 5′ end [31] and a wide variety of RNA viruses including Japanese encephalitis viruses, influenza viruses and paramyxoviruses [8], [32], while MDA5 responds primarily to poly (I:C), a dsRNA mimetic, and piconaviruses [33]. Saito *et al.* [34] and Uzri and Gehrke [35] identified the Hepatitis C virus polyuridine motif of the 3′ untranslated region (UTR) and its replication intermediate as the PAMP substrate of RIG-I. Uzri and Gehrke [35] also showed that the dengue virus full length 5′ UTR and 3′ UTR activated IFN-β to moderate levels. On the other hand, despite the innate immune system's surveillance and detection of PAMP, pathogens like DV1 still establish infection in the presence of a robust innate immune system, as viruses have evolved different mechanisms to counteract the host anti-viral response. For example, DV1 proteins such as NS2A, NS2B, NS4A, NS4B and NS5 could inhibit IFN production [36], [13], thus establishing infection in the presence of a functioning innate immune system. Other DNA and RNA viruses have been shown to comprise of IFN inhibitors as well. For example, paramyxovirus V proteins induce STAT degradation or block STAT phosphorylation, inhibiting IFN signaling [37]. Adenovirus E1A proteins and large T antigen proteins of murine polyoma virus also inhibit IFN signaling [38], [39]. NS1 protein of influenza A virus inhibits IFN-β production [40].

RIG-I-mediated IFN response was shown to play a critical role in restriction of virus infection in cultured cells. Hepatitis C virus [11], herpes simplex virus-1 and adenovirus [41] were shown to replicate to much higher titers in RIG-I mutant human hepatoma cells HUH-7.5.1 compared to wild type HUH-7 cells. Modulation of dengue virus infection by IFN has been clearly demonstrated in other studies [42], [43]. However, despite experimental evidence, the roles played by individual pattern recognition receptors in restriction of dengue virus infection are still not clearly understood. Using a microarray in our earlier study, we demonstrated IFN-related gene induction in DV1-infected cells [14]. A majority of genes, including RIG-I, MDA5 and TLR3, that were strongly up-regulated were IFN-related genes. Previous studies have shown that RIG-I and MDA5 are necessary for establishing an antiviral state against dengue virus [8]. Evidence presented in this study lends further support that both RIG-I and MDA5 are involved in induction of IFN response in dengue virus-infected cells. Interestingly, knockdown of RIG-I gene expression in HUH-7 cells in isolated stable clones showed down-regulation of both RIG-I and MDA5 expression. RIG-I and MDA5 share a limited homology in their overall primary structure [6]. The shRNA used in this study was against a target sequence of RIG-I, as described by Seth and colleagues [44] and no homology to MDA5 sequences was noted during sequence alignment. Thus, it is unclear why silencing RIG-I in HUH-7 cells would also suppress the expression of MDA5. Further investigations are required to investigate if MDA5 expression in HUH-7 cells is RIG-I-dependent or the shRNA sequence is silencing MDA5 expression in unknown ways.

The observation that RNA silencing of RIG-I and MDA5 in HUH-7 resulted in a significant increase in DV1 replication and IFN-β production raised a possibility that the third RNA sensor, TL3, may also play an important anti-DV1 role in this cell system. As the basal level of TLR3 in HUH-7 cells is low [15], its role in induction of IFN and in restriction of virus infection in this type of cells is not fully appreciated. The fact that knockdown of TLR3 by siRNA in RIG-I- and MDA5-knockdown cells (shRIG-I cells) further enhances dengue virus infection and reduces IFN-β response demonstrates that all the three pattern recognition receptors are implicated in host innate immune response to the same virus in a same infected cell, and may play a synergistic role. Although, as discussed above, DV1 produces IFN antagonists, no viral protein has been shown to interfere in the upstream sensing of viral double stranded RNA by RIG-I, MDA5 or TLR3. As these receptors are inducible in DV1-infected cells [14], the enhancement effect on viral replication by RIG-I- and MDA5-knockdown, in turn, enhances the TL3 induction and IFN-β response. This may explain why higher levels of IFN-β response were observed in RIG-I- and MDA5-knockdown cells. It also suggests that the presence of minute amounts of a specific receptor in a particular cell type would play an important restriction role. On the other hand, as these three receptors show distinct substrate specificities, it would be reasonable to assume that they may recognize different parts of the viral components. A clear advantage for the host is that the invading pathogens can be effectively captured even though mutations occurred in a certain part of the viral genome. If the virus evades one pathway of detection, another could detect and trigger innate immunity against the virus.

In summary, our data show that RIG-I, MDA5 and TLR3 synergistically activate innate immune response against DV1 infection in HUH-7 cells. Our study shows the involvement of RIG-I, MDA5 and TLR3 in innate immune response against dengue virus in the same cellular system (HUH-7). The results also show that over-expression of RIG-I or MDA5 individually induces weak IFN-β expression as compared to when both genes are over-expressed. However, over-expression of TLR3 alone could recognize DV1 and initiate strong IFN-β response. This study, thus, contributes to ongoing characterization of the innate antiviral response to dengue virus infection in cells. Understanding and identifying the molecular patterns that trigger innate immune signaling may lead to targeted and specific antiviral strategies against dengue virus infection.
